# Sec16 Defines Endoplasmic Reticulum Exit Sites and is Required for Secretory Cargo Export in Mammalian Cells

**DOI:** 10.1111/j.1600-0854.2006.00493.x

**Published:** 2006-09-27

**Authors:** Peter Watson, Anna K Townley, Pratyusha Koka, Krysten J Palmer, David J Stephens

**Affiliations:** Department of Biochemistry, University of Bristol, School of Medical Sciences, University Walk Bristol BS8 1TD, UK

**Keywords:** COP-coated vesicles, COPII-coated vesicle, endoplasmic reticulum, Golgi apparatus, protein transport

## Abstract

The selective export of proteins and lipids from the endoplasmic reticulum (ER) is mediated by the coat protein complex II (COPII) that assembles onto the ER membrane. In higher eukaryotes, COPII proteins assemble at discrete sites on the membrane known as ER exit sites (ERES). Here, we identify Sec16 as the protein that defines ERES in mammalian cells. Sec16 localizes to ERES independent of Sec23/24 and Sec13/31. Overexpression, and to a lesser extent, small interfering RNA depletion of Sec16, both inhibit ER-to-Golgi transport suggesting that Sec16 is required in stoichiometric amounts. Sar1 activity is required to maintain the localization of Sec16 at discrete locations on the ER membrane, probably through preventing its dissociation. Our data suggest that Sar1-GTP-dependent assembly of Sec16 on the ER membrane forms an organized scaffold defining an ERES.

Protein transport from the endoplasmic reticulum (ER) to the Golgi apparatus is dependent on the coat protein complex II (COPII) (1). Five core COPII components Sar1, Sec23/24 and Sec13/31 together are sufficient to generate vesicles from liposomes *in vitro*. In *Saccharomyces cerevisiae*, assembly of COPII occurs stochastically on the ER membrane. In *Pichia pastoris* (2), and in higher eukaryotes (3,4), assembly occurs at specific sites known as transitional ER or ER exit sites (ERES); the reason for this restricted localization is unknown. These sites can form de novo (5), are long-lived, and relatively static (6,7). Coat protein II vesicles bud from these sites and then rapidly uncoat with COPII subunits recycling for reassembly on the ER membrane. Mammalian homologues of the minimal COPII machinery necessary to form a COPII coat *in vitro* (Sar1, Sec23/24 and Sec13/31) have all been identified. In addition to these five proteins, the *in vivo* assembly process depends on additional proteins. Sec12 is a guanine nucleotide exchange factor (8) catalysing GDP–GTP exchange on Sar1, a small GTPase (9). This results in recruitment of Sec23, the GTPase activating protein (GAP) for Sar1 (10); Sec24, the major cargo-binding component of the coat (11); and finally Sec13 (12) and Sec31 (13) which form heterotetramers and assemble as a cuboctahedral coat on the membrane (14). A further component, Sec16, has been characterized in *S. cerevisiae* (15–17) and *P. pastoris* (18). In these organisms, Sec16 encodes a large (∼240 kD) protein, which binds to Sec23/24 and Sec13/31 (15,19). Its role appears to stabilize the GTP-bound state of Sar1 and to direct assembly of other COPII subunits (17). Notably, Sec16 is required to reconstitute GTP-dependent budding as opposed to vesicle formation in the presence of poorly hydrolyzable GTP analogues (17).

The mechanism by which COPII assembly is restricted to ERES remains unknown. In mammalian cells, Sec12 and Sar1 are localized uniformly across the ER membrane with some accumulation in to ERES [(8,9) and D. J. S., unpublished observations]. In contrast, Sec16 and the downstream COPII proteins, Sec23/24 and Sec13/31, are exclusively localized to punctate structures at, or in the immediate vicinity of, ERES. There has been some debate as to whether a Sec16 orthologue exists in mammals and the only hint to date has been the identification of a protein of ∼250 kD that cross-reacts with an antibody directed against the *S. cerevisiae* Sec16 protein (20). Here, we identify and provide initial characterization of the mammalian homologue of Sec16. We show that the human Sec16 is a peripheral membrane protein that is tightly associated with ERES, at which it recycles on and off the membrane. Overexpression or depletion of Sec16 significantly affects membrane traffic between the ER and the Golgi apparatus. We also find that Sar1-GTP is required to maintain the localization of Sec16 to ERES and find that Sar1-GTP induces assembly of Sec16 in to higher order structures. Together, our data show that Sec16 defines ERES in mammalian cells.

## Results and discussion

Sec16 has been identified in *S. cerevisiae* and *P. pastoris*. The sequences of these genes show highest homology in a central region (18). We have used this region to search the human genome sequence and have identified a previously uncharacterized transcript (KIAA0310; GenBank accession AB002308) encoding the human orthologue of Sec16. Further database searching shows that multiple splice forms of Sec16 are expressed together in the same tissue and that additional species homologues can be found in *Rattus norvegicus*, *Mus musculus*, *Bos taurus*, *Canis familiaris*, *Danio rerio, Drosophila melanogaster*, *Xenopus laevis* and other species. In all cases, the protein encoded by the gene is large (∼250 kD) and shows regions of conservation in the central and C-terminal domains that in yeast bind to Sec23/24 and Sec13/31, respectively (15,16,19). This suggests a high evolutionary conservation of Sec16 from fungi to vertebrates.

**Figure 1:**
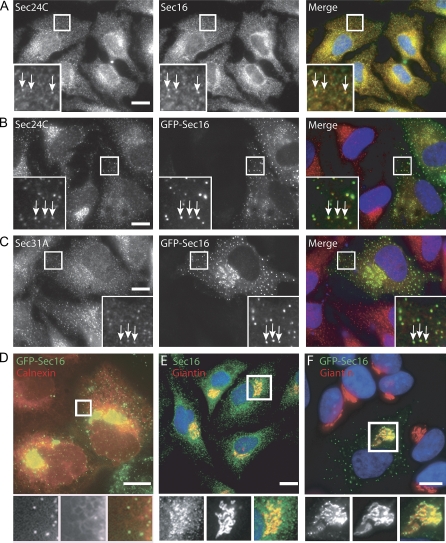
Localization of human Sec16 A) Immunofluorescence labelling of HeLa cells with anti-Sec16 (red in merge) and anti-Sec24C (green) antibodies. B and C) HeLa cells expressing EGFP-Sec16 (green in merge) at low level were methanol fixed and labelled with antibodies specific for B) Sec24C (red) or C) Sec31A (red). D) Venus-Sec16 (green in merge) puncta aligned on filaments of the ER membrane labelled with ECFP-ER (red). E and F) Endogenous Sec16 labelled using E) anti-Sec16 antibodies or F) EGFP-Sec16 in transfected cells (both green in merges) were localized in fixed cells and co-labelled with anti-giantin antibodies to reveal the close apposition of Sec16 puncta and Golgi membranes (red). Bars = 10 μm.

**Figure 2:**
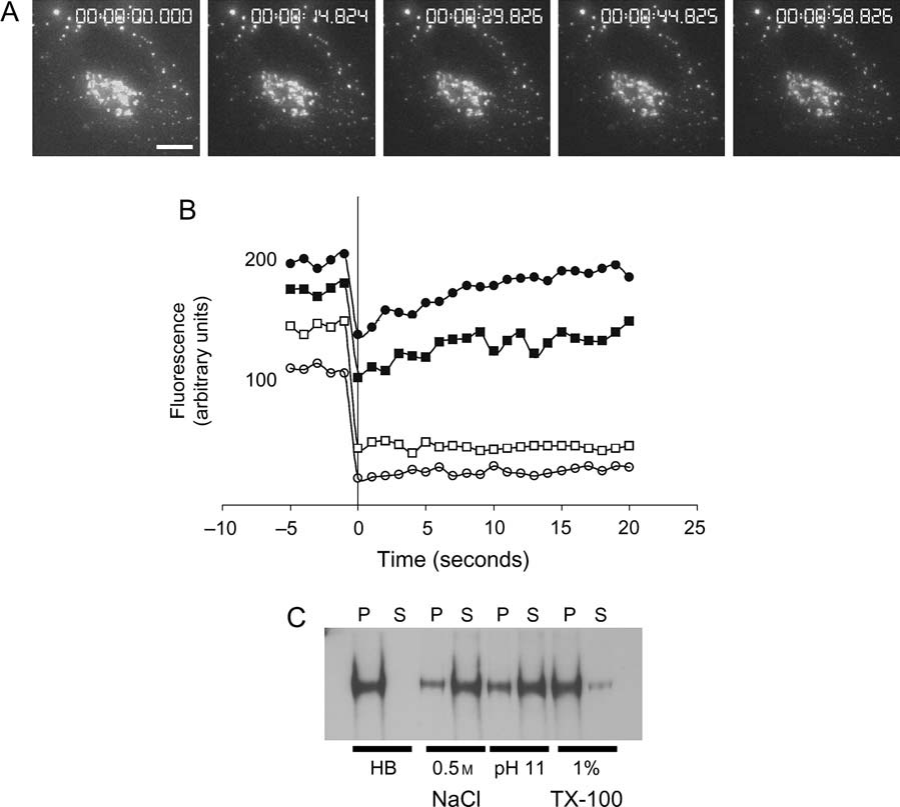
Dynamics and membrane association of Sec16 A) Time-lapse imaging of cells expressing GFP-Sec16; 10 images/second were acquired at 1 μm spacing in z and are displayed as extended focus images. In these movies of whole cell depth at 1 frame/second, we cannot identify any fast-moving punctate structures consistent with what we observe with other EYFP-tagged COPII subunits. Bar = 10 μm. B) Fluorescence recovery after photobleaching of cells expressing low (solid symbols) or high (open symbols) levels of EYFP-Sec16. Individual examples are shown, the half-life for fluorescence recovery was in the range 4.3–26.7 seconds, the mobile fraction was 80–100% (20 structures from 6 cells, two independent experiments, half-lives were calculated using Leica TCS software). Variability in expression level and the size of the mobile fraction probably reflects differences in expression level between cells. C) Ad293 cells were fractionated as described and membranes extracted with buffers [homogenization buffer containing no supplements (HB), 0.5 m NaCl, 0.1 m sodium carbonate, pH 11 or 1% TX-100] as indicated before separation into pellet (P) and supernatant (S) at 25 000 × ***g***.

Endogenous human Sec16 localize exclusively to ERES (Figure 1A). Enhanced green fluorescent protein (GFP)-tagged Sec16 expressed in HeLa cells also localize to ERES. Endogenous Sec16 (Figure 1A), or EGFP-Sec16 at low expression levels (Figure 1B, C), co-localized with COPII proteins Sec24C and Sec31A. Localization across the entire ER membrane was not observed. The EGFP-Sec16 puncta co-aligned with the underlying ER membrane (Figure 1D) as is seen for other COPII components (7). Consistent with the localization of other COPII components (for examples see (5–7)), Sec16 was accumulated in the juxtanuclear region of cells. Sec16-positive spots align very closely but are distinct from Golgi membranes (Figure 1E showing endogenous Sec16 and 1F showing GFP-Sec16). The EGFP-Sec16 showed indistinguishable dynamics from other COPII proteins (Figure 2A) (5,7). Mobility was restricted to small areas and punctate spots of EGFP-Sec16 fluorescence were long-lived and stable (Figure 2A). We did not observe any highly mobile EGFP-Sec16-positive structures even at frame rates of up to 50 frames/second. The same localization and dynamics of EGFP-Sec16 was observed in other human cell lines as well as those from rat, monkey and potoroo (data not shown). Fluorescence recovery after photobleaching showed that enhanced yellow fluorescent protein (EYFP)-Sec16 expressed at low levels recycled rapidly on and off the membrane (Figure 2B, solid symbols) as is seen for other COPII subunits (21). This is also consistent with the incorporation of *S. cerevisiae* Sec16 in to budding vesicles (16). An alternative explanation is that mammalian Sec16 cycles between a membrane-bound (ERES) pool and a cytosolic pool. At high expression levels, no recovery of fluorescence was seen suggesting that EYFP-Sec16 has irreversibly accumulated on the membrane (Figure 2B, open symbols). Cell fractionation experiments showed that endogenous human Sec16 could be eluted from membranes with high salt concentrations (0.5 m) or high pH, but not with 1% Triton-X-100 (TX-100) (Figure 2C), consistent with the properties described for *S. cerevisiae* Sec16 which is a tightly associated peripheral membrane protein (17).

**Figure 3:**
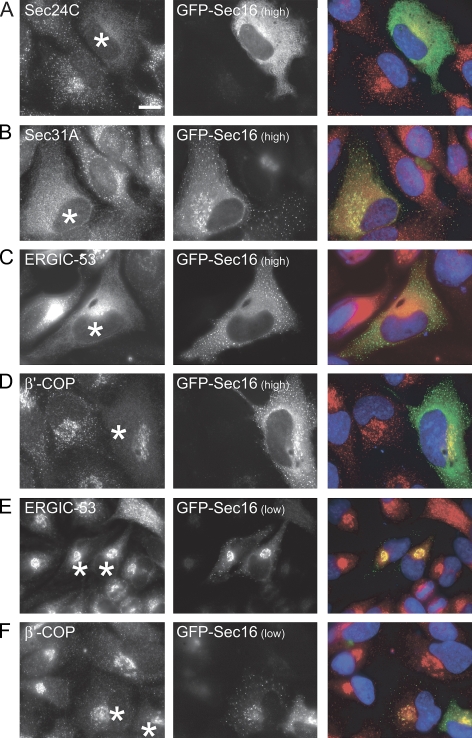
Sec16 is required in stoichiometric amounts for COPII assembly and ER-to-Golgi transport Cells expressing EGFP-Sec16 at high (punctate and cytosolic, A–D) or low levels (exclusively punctate, E and F) were labelled with antibodies directed against A) Sec24C, B) Sec31A, C and E) ERGIC-53 or D and F) β’-COP. Cells expressing EGFP-Sec16 are marked with asterisks. Note loss of Sec24C, Sec31A and β’-COP labelling in cells expressing a high level of EGFP-Sec16; ERGIC-53 is redistributed to the ER in these cells. Bars = 10 μm.

In yeast, Sec16 is required in stoichiometric amounts for transport (16). Expression of EGFP-Sec16 at low levels caused some minor displacement of Sec24C and Sec31A from ERES (especially noticeable in the juxtanuclear region, Figure 1B, C); high expression of EGFP-Sec16 caused a near complete loss of punctate staining of Sec24C and Sec31A (Figure 3A, B). High expression of EGFP-Sec16 caused complete relocalization of ER–Golgi intermediate compartment (ERGIC-53) to the ER (confirmed by double labelling with anti-calnexin antibodies, not shown) (Figure 3C). Similarly, expression of GFP-Sec16 at high levels caused a complete loss of COPI from peripheral puncta leaving only some juxtanuclear localization (Figure 3D), suggesting an inhibition of early secretory pathway function. This juxtanuclear pool of COPI co-localized with markers of the Golgi apparatus (giantin, not shown) and suggests that there is a Golgi-localized pool of COPI that does not require ongoing COPII function for its association with Golgi membranes. Low-level expression of GFP-Sec16 did not cause relocalization of ERGIC-53 (Figure 3E) or β’-COP (Figure 3F).

**Figure 4:**
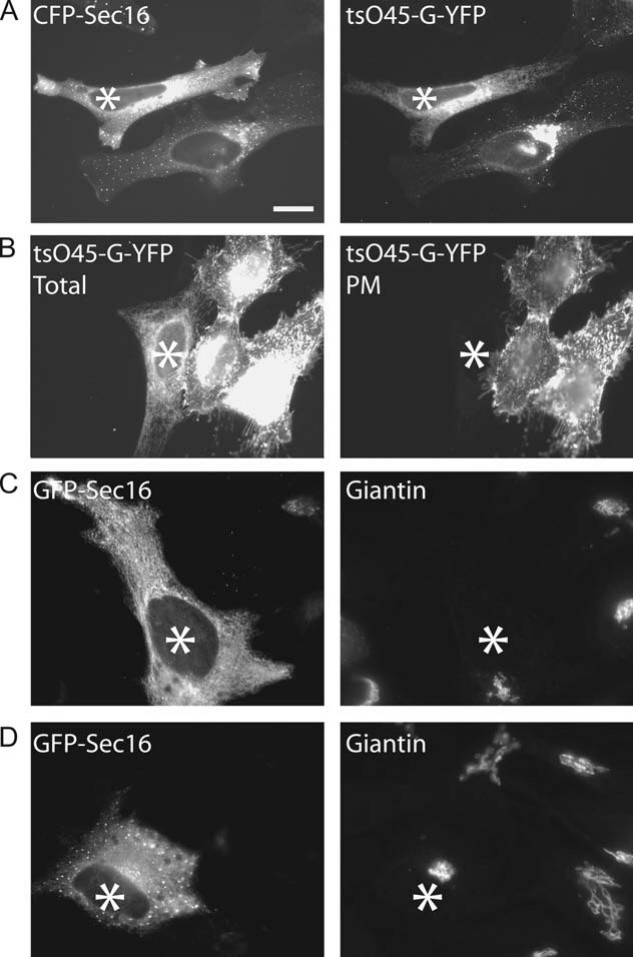
Inhibition of ER-to-Golgi transport following overexpression of CFP-Sec16 Cells expressing high levels of CFP-Sec16 were infected with Adenovirus to express tsO45-G-YFP at 39.5°C. Cells were then shifted to 32°C for A) 30 min or B) 90 min. A) In cells expressing a low level of CFP-Sec16, tsO45-G-YFP accumulates in the Golgi apparatus 30 min following shift to 32°C, while in cells expressing a high level of CFP-Sec16 (asterisk), tsO45-G-YFP remains within the ER. B) Cells incubated at 32°C for 90 min were paraformaldehyde fixed and immunolabelled to detect tsO45-G-YFP that reaches the PM. In cells expressing a high level of CFP-Sec16 (asterisk), tsO45-G-YFP remains within the ER (total) and does not reach the PM. C and D) Cells expressing GFP-Sec16 (asterisks) do not have significantly disrupted Golgi apparatus as determined by giantin localization (right hand panels). Bar (all panels) = 10 μm.

To quantitatively monitor transport of secretory cargo, we used the model cargo protein tsO45-G-YFP, which can be accumulated in the ER at 39.5°C and released as a relatively synchronous wave of cargo transport at 32°C (22,23). In cells overexpressing enhanced cyan fluorescent protein (ECFP)-Sec16 at high level, export of tsO45-G-YFP from the ER and transport to the Golgi apparatus (Figure 4A, asterisk, 30 min after shift to the permissive temperature) was completely inhibited. Note that the adjacent cell in Figure 4A expressing a low level of CFP-Sec16 (localizing exclusively to ERES) shows no inhibition of transport to the Golgi apparatus. Further transport of tsO45-G-YFP to the plasma membrane (PM) (measured 90 min after shift to the permissive temperature) was completely inhibited by high expression of CFP-Sec16 (Figure 4B). In cells expressing these high levels of GFP-Sec16, the Golgi apparatus remains intact, indicating a block in ER export rather than simply a disrupted Golgi apparatus (Figure 4C, D).

**Figure 5:**
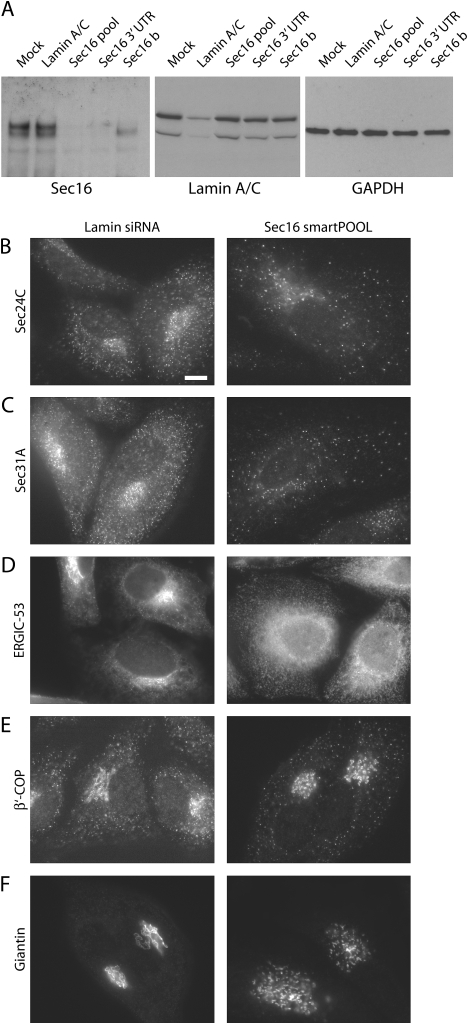
Depletion of Sec16 from human cells disrupts COPII assembly and ER-to-Golgi transport A) Depletion of Sec16 expression using siRNA. HeLa cells were transfected with siRNA duplexes targeting Sec16 [pool of four duplexes (Sec16 pool), lamin A/C, a duplex targeting the 3′ UTR of Sec16 (Sec16 3′ UTR) and a further Sec16 duplex targeting the coding region (Sec16 b)]. Cell lysates were then immunoblotted for Sec16 (250 kD), lamin A/C (84 kD) or GAPDH (as a loading control, 40 kD) as indicated. Note that the duplex termed Sec16b only depletes a subset of splice forms of Sec16. Cells depleted of Sec16 using a pool of four siRNA duplexes were methanol fixed and labelled with antibodies specific for B) Sec24C, C) Sec31A, D) ERGIC-53, E) β’-COP and F) giantin as indicated. Bar (all panels) = 10 μm.

To determine the requirement for Sec16 in ERES organization and membrane traffic from the ER, we depleted the protein from cells using RNA interference. Immunoblotting with antibodies directed against Sec16 showed that we were able to efficiently deplete Sec16 with a pool of four duplexes as well as with individual duplexes (Figure 5A). One duplex depleted only one of the two bands observed, suggesting that it is not effective against all splice forms that are expressed in HeLa cells. The role of these different splice forms remains unclear. Depletion of lamin A/C was used as a control and none of the small interfering RNA (siRNA) duplexes used caused any changes in expression of glyceraldehyde 3-phosphate dehydrogenase (GAPDH) (Figure 5A). Using a pool of four siRNA duplexes targeting Sec16 (Sec16 smartPOOL), we found that depletion of Sec16 resulted in fewer punctate spots of Sec24C (Figure 5B) and Sec31A (Figure 5C) within the cell, particularly significant within the juxtanuclear area. Quantification of the number of spots in the periphery of cells shows a decrease from 25 ± 10/10 μm^2^ in lamin A/C-depleted cells to 4 ± 3/10 μm^2^ in Sec16-depleted cells (*n* = 100 cells from three independent experiments). Similarly, ERGIC-53 was seen to be localized to the ER (Figure 5D), fewer punctate spots of COPI were visible in the periphery (Figure 5E), and giantin localization was more dispersed and puncta compared with its typical ribbon-like appearance (Figure 5F). Ninety-six per cent of cells show these distributions following depletion of Sec16, likely a reflection of siRNA transfection (or depletion) efficiency. The same results were obtained when siRNA experiments were performed both with a pool of four duplexes as well as individual duplexes included in the pool and other independent duplexes, including the one targeting the 3′ untranslated region (UTR) of the Sec16 messenger RNA (see *Materials and Methods*).

**Figure 6:**
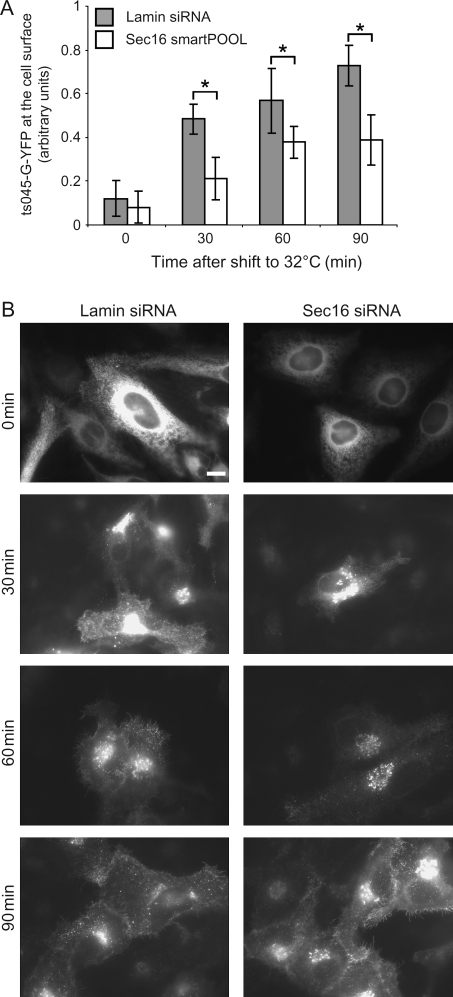
A) Cells depleted of either Lamin A/C or Sec16 were infected with Adenovirus to express tsO45-G-YFP at 39.5°C followed by incubation at 32°C for the times indicated Cells were depleted of either Lamin A/C (filled bars) or Sec16 (using a pool of four duplexes, open bars). The amount of tsO45-G-YFP reaching the cell surface was quantitated for each time-point. tsO45-G-YFP shows a significant (anova, * p = 0.04) delay in reaching the PM in cells depleted of Sec16. B) Seventy-two hours after transfection with siRNA duplexes, cells were infected with Adenovirus to express tsO45-G-YFP at 39°C followed by shift to 32°C for the times indicated and quantification of the amount of tsO45-G-YFP reaching the PM. Bar (all panels) = 10 μm.

Transport between the ER and the Golgi apparatus was also significantly disrupted following siRNA depletion of Sec16. In a quantitative assay of tsO45-G transport to the PM, the amount of protein reaching the PM following depletion of Sec16 is approximately 50% for the 30- and 90-min time-points (Figure 6A). The statistical significance was confirmed using anova (asterisks, p < 0.04); differences within an individual experiment were greater than those across the three independent experiments that have been pooled for the data in Figure 6A. This inhibition of transport results from a delay in ER export and accumulation in the Golgi apparatus, shown by the localization of the protein during the assay (Figure 6B). Note the accumulation of the protein in the Golgi apparatus in Sec16-depleted cells 30 min after shift to the permissive temperature (Figure 6B). We were unable to deplete Sec16 protein levels further with any of the duplexes tested, possibly due to high stability of the protein or reflecting an essential function. Multiple immunoblots show that depletion is a maximum of 80%. Our inability to further deplete Sec16 probably underlies the incomplete inhibition of transport that we observe in the localization and transport assays. Depletion using siRNA is not the same as a gene knockout in yeast (in which all of the protein is absent) and this could explain the lack of complete inhibition of transport seen here.

**Figure 7:**
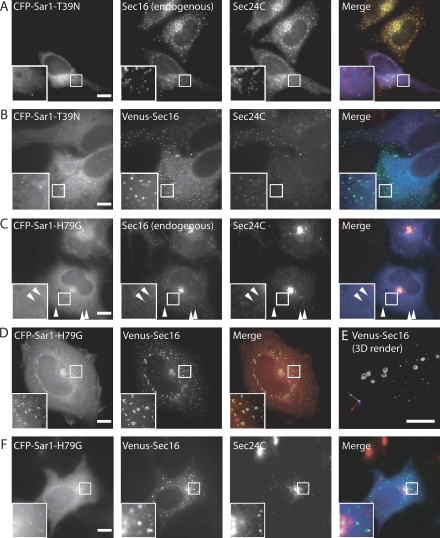
Sar1-GTP mediates assembly of Sec16 at ERES A) Cells expressing ECFP-Sar1-T39N (GDP restricted, blue in merge) were labelled with antibodies to detect endogenous Sec16 (green) and Sec24C (red). The majority of punctate Sec16 localization is lost; those few punctate spots of Sec16 that remain also label with anti-Sec24C antibodies, as they do in non-transfected cells. B) Cells were transfected with both ECFP-Sar1-T39N (blue in merge) and Venus-Sec16 (green) and immunolabelled for endogenous Sec24C (red). C) Cells transfected with ECFP-Sar1-H79G (blue in merge) were labelled for endogenous Sec16 (green) and Sec24C (red). Sec24C is not present on Sec16-positive structures (arrowheads) D) 3D imaging of paraformaldehyde-fixed cells expressing both ECFP-Sar1-H79G (green in merge) and Venus-Sec16 (red) shows that both proteins localize to large curved and round structures. E) 3D rendering of deconvolved stacks from cells expressing both ECFP-Sar1-H79G and Venus-Sec16 reveal that these objects often form near complete spheres. F) Immunolabelling of ECFP-Sar1-H79G (blue in merge) and Venus-Sec16 (green) labelled structures shows that they do not contain Sec24C (red). Boxed regions show threefold enlargements of selected areas. Representative images are shown from four independent experiments in which a total of 400 cells were inspected visually. All bars = 10 μm.

In *S. cerevisiae*, Sec16 is recruited to neutral liposomes in a Sar1- and GTP-dependent manner (17); however, in *P. pastoris*, expression of a GDP-restricted form of Sar1 does not result in a loss of Sec16 localization (18). Here, we found that in human cells, expression of Sar1-T39N [a GDP-restricted form (9)] resulted in a near complete loss of Sec16 and Sec24 from punctate structures (Figure 7A, also Sec31, not shown). Note that Sec16 and Sec24C co-localize in non-transfected cells (Figure 7A); in Sar1-T39N-transfected cells, those puncta that remain contain both Sec16 and Sec24C (albeit very faintly, Figure 7A, box), suggesting that they are indeed *bona fide* ERES. Quantification shows that >90% of Sec24 was lost from these sites (*n* = 200 ERES from four independent experiments). Expression of ECFP-Sar1-T39N together with Venus-Sec16 resulted in localization of Venus-Sec16 to punctate spots but displacement of >95% of Sec24C from the membrane [Figure 7B (*n* = 200 ERES from four independent experiments)]. Again the presence of very faint Sec24 localization to these sites strongly suggests that they are indeed *bona fide* ERES. These data suggest that, at endogenous levels of expression, Sec16 requires Sar1-GTP for association with ERES; however, when overexpressed, Venus-Sec16 can assemble at ERES independent of GTP-loading of Sar1. We interpret this as an inherent capacity of the protein to self-assemble that is exaggerated on overexpression. An alternative explanation is that the higher levels of Sec16 resulting from overexpression are able to bind to pre-existing ERES more efficiently. This result is in direct contrast to the situation in *P. pastoris*, where expression of Sar1-T34N does not cause a loss of Sec16 or Sec23/24 from ERES (18). While this might reflect a difference between these species, it could also be explained by differences in expression level. Previous work has shown that GTP is required with Sar1 to recruit *S. cerevisiae* Sec16 to dioleoylphosphatidylcholine/dioleolyphosphatidylethanolamine (DOPC/DOPE) liposomes (although Sec16 binds independently to other liposomes) (17). As Sec16 does not become recruited to the entire ER membrane (where Sar1 will become activated by Sec12), the simplest interpretation of our data is that Sar1-GTP is required to maintain the association of Sec16 with ERES, perhaps by preventing its dissociation. Overexpression of GFP-Sec16 overcomes this, such that ERES are maintained even in the presence of Sar1-T39N. These data further support the idea of self-assembly of higher order Sec16 structures.

Following expression of a GTP-restricted form of Sar1 (Sar1-H79G), endogenous Sec16 as well as Sec24 (Figure 7C) and Sec31 (not shown) accumulated on membranes in a juxtanuclear area [as has been described before e.g. see (24–26)]. In addition to localizing to these juxtanuclear structures, Sec16 also remained associated, albeit at reduced levels, with peripheral puncta (Figure 7C, arrowheads). These Sec16 peripheral puncta were not labelled with antibodies against Sec23/24 (Figure 7C) or Sec13/31 but were still associated with the ER membrane (data not shown). More than 50 cells were inspected in each of the three independent experiments. Thus, Sec16 localizes to ERES independent of Sec23/24 and Sec13/31 and is therefore a *bona fide* marker of these sites in a way that other COPII proteins are not. This experiment is consistent with recent quantitative data showing that the majority (∼80%) of COPII label is associated with free vesicles and tubules and not with the ERES membrane (27). Our interpretation is that in the presence of Sar1-H79G, those structures that move to the centre of the cell are these free vesicles and tubules (presumably driven to the centre by a dynein–dynactin-based process (22,28)), while a significant amount of Sec16 remains associated with the ERES. This reinforces the idea that Sec16, rather than the other previously identified mammalian COPII homologues, defines an ERES, while Sec23/24 and Sec13/31 define COPII-coated vesicles.

Strikingly, when imaging cells expressing both ECFP-Sar1-H79G and Venus-Sec16, we observed the formation of large ring-like structures visible in the cell periphery that were strongly labelled with Venus-Sec16 and also, more weakly, with ECFP-Sar1-H79G (Figure 7D). Three-dimensional rendering revealed these structures to be curved or near spherical (Figure 7E). Immunofluorescence showed that these structures do not contain Sec23/24 or Sec13/31 (Figure 7F) but do align with the underlying ER membrane (not shown). These structures were not visible following expression of ECFP-Sar1-H79G alone but only appeared following co-expression of both ECFP-Sar1-H79G and Venus-Sec16. Their size is always limited to a diameter of <2 μm. These higher order structures likely reflect an exaggerated state but implicate Sar1-GTP in the organization and/or maintenance of Sec16-containing structures. However, this assembly does not occur all across the ER membrane but at restricted sites. As shown above, these structures do not contain Sec23/24 or Sec13/31 and so probably represent a hyper-assembled state of Sec16 that is not able to direct further assembly of COPII. These structures contain a significant amount of Sec16 and a small amount of Sar1; it is possible that Sec16 stabilizes a curved membrane domain generated by Sec16-mediated concentration of Sar1-GTP (29,30). Alternatively, Sec16 might induce curvature of the membrane in its own right. Similar curved structures have been seen in electron micrographs of cells expressing Sar1-GTP alone (4). Sec16 is known to stabilize the GTP-bound form of Sar1 to facilitate productive budding (17). Furthermore, at high concentrations, Sar1-GTP can induce curvature of membranes and GTP hydrolysis drives vesicle fission (29,30). We propose that Sec16 serves to concentrate and stabilize Sar1-GTP, such that this membrane deformation and scission occurs only at ERES rather than all across the ER where Sar1 is less concentrated.

**Figure 8:**
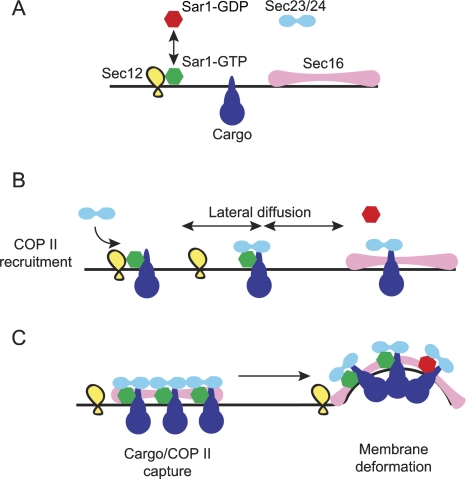
Model to describe Sec16 function in mammalian cells A) The small GTPase Sar1-GDP (red) can be recruited to the ER membrane by Sec12 (its guanine nucleotide exchange factor, yellow). Cargo proteins (blue) diffuse through the ER membrane and Sec16 assemblies (light magenta) are present at ERES. B) Sar1-GTP can bind to some cargo molecules and recruits Sec23/24 (cyan). Complexes of Sar1-GTP-cargo-Sec23/24 can diffuse within the plane of the ER membrane. GTP turnover on Sar1 could lead to its dissociation here while cargo and Sec23/24 remain associated. C) Sec16 assemblies could act to capture and stabilize cargo-Sec23/24 complexes either in the presence or absence of Sar1. Sec16 acts to concentrate Sar1-GTP-cargo-Sec23/24 pre-budding complexes such that membrane deformation occurs. See text for further details.

We conclude that in human cells, Sec16 is required for secretory cargo traffic from the ER to the Golgi apparatus. Sec16 and Sar1 appear to have an interdependent relationship, in which the localization of Sec16 to the ER membrane depends on Sar1-GTP. In addition, the stable accumulation of Sar1-GTP–Sec23/24-cargo complexes also requires Sec16. We therefore propose a model (Figure 8) as follows. Sar1-GDP (red) can be activated to the GTP-bound state (Sar1-GTP, green) by Sec12 (yellow) at sites all across the ER membrane (8) (Figure 8A). This would allow continued ‘sampling’ of membrane proteins (e.g. cargo, blue) for inclusion in budding vesicles and concomitant binding of Sec23/24 (cyan) to both Sar1-GTP and transmembrane cargo. Cargo itself can stabilize the GTP-bound form of Sar1 (21,31,32), which would probably result in recruitment of Sec23/24, which can bind to both Sar1-GTP and cargo directly (Figure 8B). Sec12 activates Sar1 with a 10-fold higher turnover rate than the GAP activity of Sec23/24 (33), which suggests that these complexes would have some stability. This could result in direct recruitment of Sec16 (light magenta), or more likely given the discrete localization of Sec16 to ERES, diffusion of these cargo-Sar1-GTP complexes within the ER membrane, such that they would encounter a Sec16 assembly and be ‘captured’ (Figure 8C). Thus, Sar1-GTP is not required for recruitment of Sec16 per se but is required to maintain its assembly at ERES. Sec16 can bind Sec23/24 independently of Sar1 (17) and so could act as a reservoir for these COPII subunits. The final budding step requires Sar1-GTP, such that curvature is induced for vesicle formation (29,30). Turnover of GTP by Sar1 could result in disassembly of these complexes. In yeast, Sec16 does not affect the GTPase rate of Sar1 (17), and so induction of curvature would require some mechanism to maintain a pool of Sar1-GTP. Sec12 does not bind Sec16 (34) and is not incorporated in to the nascent vesicle and so might not be able to perform this function at these sites. There are two obvious possibilities for the maintenance of Sar1-GTP here. One is that the assembly of Sec16, Sar1-GTP, Sec23/24 and cargo is sufficient to induce membrane curvature (i.e. the onset of vesicle formation), such that Sec13/31 becomes recruited. Sec13/31 in itself forms cages (14), suggesting that it might prefer to adopt a curved structure rather than a planar lattice. Sec13/31 further stimulates the GTPase rate of Sar1 above that of Sec23/24 alone (35); such that Sar1 dissociates from the fully coated, budded vesicle and uncoating is triggered. Alternatively, other components might modulate the GTPase cycle of Sar1 in the same way that Sed4 has been proposed to function in *S. cerevisiae* (34,36). We have not been able to identify a mammalian Sed4 homologue by sequence alignment but this does not preclude its existence; another unrelated protein could also perform this function.

In summary, we have identified the mammalian orthologue of Sec16 and show that it plays a key role in COPII function. Our data indicate that Sec16 has the capacity to assemble on the ER membrane and that its persistence on the membrane is controlled by the GTPase activity of Sar1. We hypothesize that assembly of Sec16 co-ordinates the assembly of the COPII pre-budding complex of Sar1, Sec23/24 and thereby provides the spatial coordination for rapid, productive COPII budding events.

## Materials and methods

### Cell culture and transfection

All cell lines [HeLa (ATCC CRL-2), NRK (ATCC CRL-6509), Hs68 (ATCC CRL-1635), PtK2 (ATCC CCL-56), Vero (CCL-81) or Ad293 (Stratagene, La Jolla, CA, USA)] were grown in DMEM supplemented with 10% foetal calf serum. All experiments were performed in HeLa cells except where indicated. Cells were transfected with plasmid DNA using Fugene6 (Roche, Lewes, UK) according to the manufacturers instructions. Small interfering RNA transfection was achieved using a modified calcium phosphate procedure as described in reference (37).

### DNA cloning

The full-length complementary DNA encoding human Sec16 (KIAA0310) was obtained from the Kazusa DNA Research Institute, Chiba, Japan (www.kazusa.or.jp/huge) (38). Following amplification by polymerase chain reaction, it was cloned in frame to generate fusion proteins with EGFP, EYFP, ECFP (all from Clontech, Mountain View, CA, USA) or Venus fluorescent protein [termed Venus throughout the manuscript (39)].

### Antibody generation

Polyclonal antibodies directed against Sec16 were raised in sheep by PTU/BS (Penicuik, UK) by immunization with keyhole limpet haemocyanin-conjugates of the following peptide sequences, which include an N-terminal cysteine for coupling (CRREHSAFGDRPEKRD and CELPSSRPEGSQGGEL). Antibodies were affinity purified using Sulfolink (Perbio, Cramlington, UK) immunofluorescence and cell imaging. Fixation, labelling and imaging were as described (5). In addition, experiments were confirmed using a rabbit polyclonal anti-KIAA0310 antibody (BL2467) from Bethyl Laboratories (Montgomery, TX, USA).

### Cell imaging

Cells were imaged using an Olympus (London, UK)/TILL Photonics (Graefelfing, Germany) widefield imaging system (Olympus 100× 1.4 NA lens, 1× binning) as previously described (5). Images were imported in to Adobe Photoshop and resized without resampling and enlargements (where indicated) were produced with bicubic interpolation. Images in Figure 7D show brightest point merges of image stacks acquired at 0.1-μm intervals over 2 μm. Images were deconvolved by iterative deconvolution using Volocity software (Improvision, Coventry, UK) with a calculated point spread function for each fluorophore. The 3D-rendered image in Figure 7E was generated using Volocity with a fluorescence rendering engine. Fluorescence recovery after photobleaching was performed using a Leica SP2 AOBS system with HCX PL APO lbd.BL 63× 1.4 NA ultraviolet-corrected oil immersion objective scanning a 512 × 64 pixel area at 400 Hz with twofold averaging and a zoom setting of 2. Excitation of YFP was done using the 514-nm line of an argon laser. Fluorescence emission was acquired between 520 and 565 nm. Photobleaching were performed at 37°C, live-cell imaging shown in Figure 2 was performed at 20°C. The time-lapse sequence in Figure 2 as well as images and quantification of tsO45-G-YFP transport assays were acquired using an Improvision 3DM system comprising an IX-81 microscope (Olympus Microscopes, London, UK), with ASI PZ-2000 XYZ stage (Applied Scientific Instruments, Eugene, OR, USA) DG-4 illumination system (Sutter, Novato, CA, USA) with Brightline ‘Pinkel’ filter sets with single band exciters and multi-band dichroic and emission filters (Semrock, Rochester, NY, USA). For Figure 2A, images were acquired using a Hamamatsu 9100-12 EMCCD (1× binning but cropped to region of interest shown).

### tsO45-G-YFP transport assays

Cells were infected with Adenovirus engineered to express tsO45-G-YFP (40) (kindly provided by Patrick Keller and Kai Simons; MPI-CBG, Dresden, Germany) for 30 min at 37°C and then washed and transferred to 39.5°C for 16 h. Transport assays were carried out as described in (28) by shifting cells to 32°C for the times indicated. The amount of tsO45G-YFP that had reached the PM was quantified using paraformaldehyde fixed, non-permeabilized cells and anti-VG, an antibody that detects a lumenal/surface exposed epitope of the protein. Images were acquired using a Hamamatsu Orca-ERG CCD camera (Hamamatsu, Welwyn Garden City, UK). Cells expressing very high levels of tsO45-G-YFP (>3000 greyscale units on a 12-bit scale) or very low levels (<500 greyscale units) were excluded from the quantification. Fluorescence intensities were quantified manually using Volocity (Improvision).

### siRNA depletion

Four pooled siRNA duplexes directed against Sec16 were obtained from Dharmacon (Lafayette, CO, USA) (sense oligonucleotide sequences: CUAAUCAGCCUGCUAAUUU; GGACGGAAGCCUAUGAGUA; GCGGUCAGCUUAUCAAAGU; GGAGAGCUUUCGCGCUGUA). In addition, two further independent duplexes (Sec16 3′ UTR: GCAGCUCUGGAACUUAGUA and Sec16 b: CCAUUCCAGUCAUCAGGAA) were designed and ordered from MWG Biotech (Ebersberg, DE, USA). All experiments were repeated with at least two individual duplexes in addition to the pool of four Dharmacon duplexes together. Lamin A/C was depleted as a targeted control using the following duplex: CUGGACUUCCAGAAGAACA. Cells were transfected as described in (37), a protocol that results in >98% transfection efficiency of HeLa cells, and incubated for 72 h before analysis. Depletion was confirmed by immunoblotting. Cells were lysed in buffer containing 0.5 m NaCl and the postnuclear supernatant separated by gel electrophoresis on Tris-acetate (for Sec16) or Bis-Tris gels (for lamin A/C and GAPDH) (Invitrogen, Paisley, UK) and immunoblotted using antibodies against Sec16 (this manuscript), or lamin A/C (Cell Signaling Technology, Danvers, MA, USA) and anti-sheep or anti-rabbit antibodies conjugated to horseradish peroxidase; GAPDH expression, monitored as a loading control using an horseradish peroxidase-conjugated anti-GAPDH antibody (Abcam, Cambridge, UK). Blots were developed using ECL (GE Healthcare Biosciences, Little Chalfont, UK).

### Cell fractionation

Ad293 cells were grown to confluence on a 15-cm culture dish, scraped in to ice-cold homogenization buffer (HB: 50 mm HEPES pH 7.4, 0.25 m sucrose) and homogenized by 12 passes through a ball bearing homogenizer (Isobiotec, Heidelberg, Germany) with 10 μm clearance. This crude homogenate was then centrifuged at 25 000 × ***g*** and the pellet resuspended in 1 mL HB. Aliquots (0.2 mL) were then incubated for 60 min with constant mixing at 4°C in the presence of either HB, HB-containing 0.5 m NaCl, HB-containing 0.1 m sodium carbonate pH 11 or HB-containing 1% TX-100. Samples were then centrifuged for 30 min at 25 000 × ***g*** and supernatant and pellet fractions separated by SDS–PAGE (3–8% Tris-acetate gradient gels, Invitrogen), transferred to nitrocellulose and probed with anti-Sec16p.
